# Blueprint for a minimal photoautotrophic cell: conserved and variable genes in *Synechococcus elongatus *PCC 7942

**DOI:** 10.1186/1471-2164-12-25

**Published:** 2011-01-12

**Authors:** Luis Delaye, Carmen M González-Domenech, María P Garcillán-Barcia, Juli Peretó, Fernando de la Cruz, Andrés Moya

**Affiliations:** 1 Institut Cavanilles de Biodiversitat i Biologia Evolutiva, Universitat de València, Valencia, Spain; 2 Departamento de Ingeniería Genética CINVESTAV-Irapuato, Km. 9.6 Libramiento Norte, Carretera Irapuato-León, 36821 Irapuato, Guanajuato, México; 3 Facultad de Farmacia, Universidad de Granada, Granada, Spain; 4 Departamento de Biología Molecular e Instituto de Biomedicina y Biotecnología de Cantabria (IBBTEC), Universidad de Cantabria-CSIC-IDICAN, Santander, Spain; 5 Departament de Bioquimica i Biologia Molecular, Universitat de València, València, Spain; 6 Departament de Genètica, Universitat de València, València, Spain

## Abstract

**Background:**

Simpler biological systems should be easier to understand and to engineer towards pre-defined goals. One way to achieve biological simplicity is through genome minimization. Here we looked for genomic islands in the fresh water cyanobacteria *Synechococcus elongatus *PCC 7942 (genome size 2.7 Mb) that could be used as targets for deletion. We also looked for conserved genes that might be essential for cell survival.

**Results:**

By using a combination of methods we identified 170 xenologs, 136 ORFans and 1401 core genes in the genome of *S. elongatus *PCC 7942. These represent 6.5%, 5.2% and 53.6% of the annotated genes respectively. We considered that genes in genomic islands could be found if they showed a combination of: a) unusual G+C content; b) unusual phylogenetic similarity; and/or c) a small number of the highly iterated palindrome 1 (HIP1) motif plus an unusual codon usage. The origin of the largest genomic island by horizontal gene transfer (HGT) could be corroborated by lack of coverage among metagenomic sequences from a fresh water microbialite. Evidence is also presented that xenologous genes tend to cluster in operons. Interestingly, most genes coding for proteins with a diguanylate cyclase domain are predicted to be xenologs, suggesting a role for horizontal gene transfer in the evolution of *Synechococcus *sensory systems.

**Conclusions:**

Our estimates of genomic islands in PCC 7942 are larger than those predicted by other published methods like SIGI-HMM. Our results set a guide to non-essential genes in *S. elongatus *PCC 7942 indicating a path towards the engineering of a model photoautotrophic bacterial cell.

## Background

The design and implementation of simpler biological systems is relevant for biotechnological applications, and as a way to address basic biological questions. In principle, simplified versions of extant cells should be easier to understand and engineer towards a variety of tasks like production of biomolecules or exploitation of metabolic or physiological process. Besides, the search for simpler cells through genome reduction might help to identify novel species-specific essential genes under a set of defined conditions. This in turn will lead to improvements in our understanding of the basic machinery of cells, and ultimately will shed light into the minimal and sufficient features required for life [1].

Genomes of free living bacteria often contain a plethora of genes necessary to face a changing and unpredictable environment. For instance, it was shown that among different strains of the marine picocyanobacteria *Synechococcus *sp. only between 52 - 67% of the genes in each strain (~1,572 gene families) were shared among all sequenced genomes [2]. A similar situation was shown for *Prochloroccocus marinus *where only 40 - 67% of the genes (1,250 gene families) were shared by all sequenced strains [3]. While genes from the core genome (i.e., the set of genes conserved among all strains) code for essential genes, the set of variable genes might be composed of a) genes necessary for living in particular micro-environments (to which each strain is adapted); b) genes relatively neutral to cell fitness that might have arrived by HGT; and c) selfish genetic elements [4]. Although some of these variable genes might be necessary to survive in nature, they probably are unnecessary under laboratory or other artificial conditions. They can even constitute barriers that obstruct human manipulation of the organism for biotechnological or other purposes. The concept of a *tabula rasa *genome [5] implies that by eliminating the set of variable genes, a reduced (and optimized) genome with the necessary functions to sustain a cell would be obtained.

Identification of dispensable genes in genomes is often carried out through comparative analysis between strains (i.e., genes present in some but absent in other strains are classified as dispensable). This approach was performed successfully for *Escherichia coli*, where a reduced version of its genome with robust metabolic performance was engineered [5]. By comparative genomics, Pósfai and co-workers [5] identified segments of the chromosome of *E. coli *K-12 MG1655 that were absent in five other *E. coli *strains. These genomic islands (GI), putatively code for non-essential genes. By focusing initially on large segments or GIs coding for IS-elements, they deleted approximately 15% of the genome of *E. coli*. Surprisingly, the resulting *E. coli *strain, endowed with this reduced genome, showed a number of emergent properties, including more rapid growth rate, increased genome stability and high electroporation efficiency.

*Synechococcus elongatus *PCC 7942 is an obligate photoautotrophic fresh-water cyanobacteria described initially by von Nägeli [6]. Two strains from *S. elongatus *(PCC 6301 and PCC 7492) have been sequenced to date [7]. With a genome of approximately 2.7 Mb and two plasmids (8 kb and 46 kb) *S. elongatus *was the first cyanobacteria demonstrated to be transformable by exogenously added DNA [8]. It is a model for the circadian clock in prokaryotes [9]. Large scale inactivation experiments were performed to identify loci that are important for circadian rhythms of gene expression [10]. Its biological properties, like autotrophy, transformability, relatively rapid growth rate and easiness of cultivation, make this organism an ideal candidate for biotechnological applications that require a photosynthetic organism. Here we carry out a bioinformatic analysis of the *S. elongatus *genome and identify regions coding for putatively dispensable or essential genes. This work constitutes a first step towards the engineering of a reduced genome for a model autotrophic cyanobacteria.

## Results and Discussion

### Phylogenomic analysis

**Figure 1:**
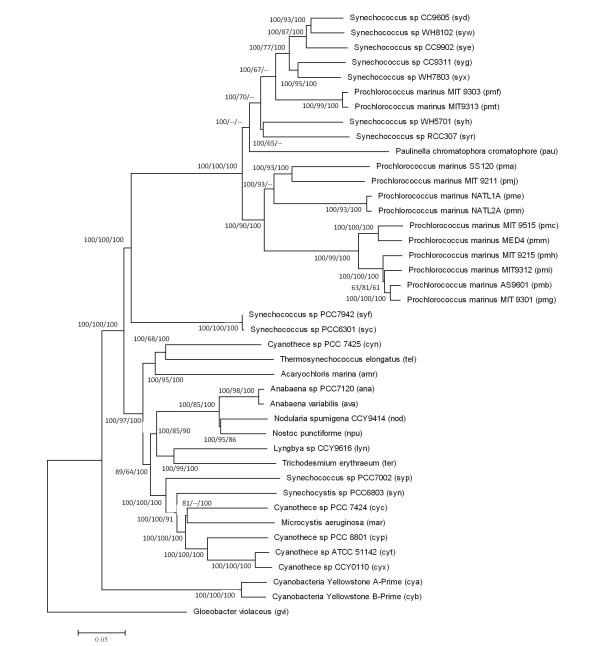
**Phylogenomic tree of cyanobacteria**. Neighbor-Joining tree reconstructed from a matrix of 231 concatenated protein families conserved among sequenced cyanobacteria (63,065 amino acid positions, Poisson distance correction). Bootstrap values (100 replications) for Neighbor-Joining and Parsimony, and quartet puzzling support values for Maximum-Likelihood are shown in nodes in the following order: Neighbor-Joining/Maximum-Likelihood/Parsimony.

To better evaluate the results from a comparative genome analysis, we reconstructed a phylogenetic tree from available cyanobacterial genomes. Reciprocal best BLAST hit searches were performed among 41 cyanobacterial genomes, revealing 473 protein families of putative orthologous genes. From them, we selected 231 families belonging to the set of conserved proteins identified by [11]. After aligning individual protein families and trimming for poorly aligned regions, we concatenated all sequences to get a final matrix of 63,065 amino acid residues. A Minimum-Evolution tree was reconstructed from this matrix (Figure 1). The resulting tree is in general agreement with other phylogenomic trees [11-13] and with 16S rRNA trees [14]. The branching of *Synechococcus *sp. WH5701 within *Prochlorococcus *clade has been observed in other phylogenomic analyses [13]. As shown in Figure 1, while *S. elongatus *is the sister group of marine picocyanobacteria (*Prochlorococcus marinus *and *Synechococcus *sp.) it is not closely related to any other cyanobacterial lineage in the dataset.

### Detection of genomic islands

Genomic comparison between different strains of a single species is the most direct approach to identify conserved and variable regions in genomes. This approach was successfully applied to identify deletable genes in *Escherichia coli *[15]. However, as previously reported for *S. elongatus *[7], genomic comparison of PCC 7942 and PCC 6301 strains revealed that this approach was not useful for these genomes due to lack of large genomic differences. The single major difference between both genomes was an inversion comprising ~188.6 kb located from nucleotides 1,704,219 to 1,515,607 in PCC 7942. Smaller differences corresponded to single nucleotide polymorphisms and two small deletions in PCC 7942 relative to PCC 6301. Also, differences in protein coding sequences among genomes reflected differences in annotations rather than differences at the level of DNA (data not shown). This low level of divergence was also evident from the short branches separating both strains in Figure 1.

To deal with the lack of available genomic diversity among sequenced *Synechococcus elongatus *strains, we relied on a combination of methodologies to seek for horizontally transferred genes (HTG). In the first place, we looked at three features that could be indicative of a xenology, namely: a) genes having few (or none) copies of a signature motif (see below) in combination with an unusual codon usage; b) genes having an unusual phylogenetic relationship; and c) genes with an unusual G+C content. We classified as xenologs all genes sharing at least two of the previous characteristics. Second, we also included as xenologs those genes sharing a predicted operon with previously identified xenologous genes if having one of three features mentioned above. Third, we reviewed information on IslandPath and IslandViewer databases to compare the genes contained in these databases, known to be mobile, against our set of identified xenologs, to look for genes that might have passed undetected by our methods. Fourth, we compared the genome of PCC 7942 with metagenomic sequences from a microbialite to look for regions of no coverage that could suggest genomic islands. Finally, the number and localization of ORFans was also investigated. The results are detailed next.

#### Unusual codon usage and signature motif HIP1

**Figure 2:**
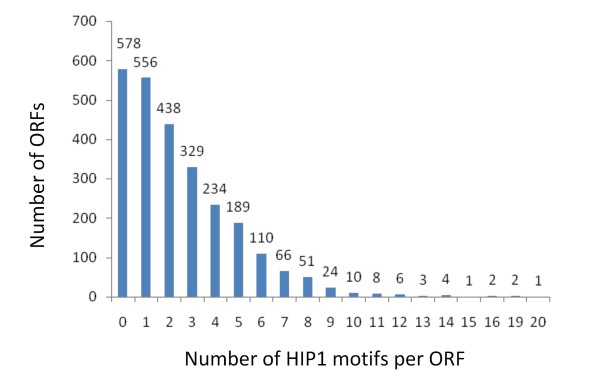
**Distribution of HIP1 motifs per ORF in PCC 7942**. The X-axis shows the categories of genes, according to their number of HIP1 motifs. The Y-axis shows how many PCC 7942 genes belong to each category. The distribution is reminiscent of a random process, with most ORFs having none or a few copies of HIP1, and a few ORFs having several ones.

**Figure 3:**
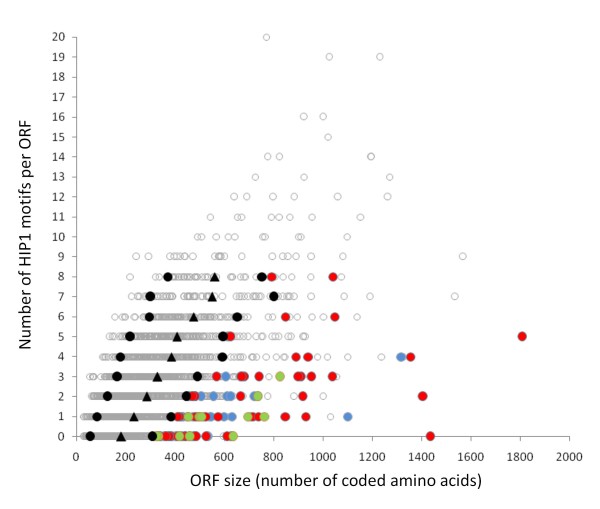
**Distribution of HIP1 motifs per ORF in PCC 7942 versus ORF length**. Each dot in the figure represents one gene from the PCC 7942 genome. They are sorted by their number of coded amino acids (X-axis) and by the number of HIP1 motifs in their coding sequence (Y-axis). Black triangles show the mean of the size distribution for each category (0 HIP1 motifs, 1 HIP1 motifs... etc); black circles indicate the positions separated by one standard deviation of the mean; green dots represent ORFs with a codon usage reminiscent of highly expressed genes (PHX); blue dots, genes with codon usage similar to (but not as pronounced as) highly expressed genes (PX); and red dots putative alien genes (PA); gray dots, other genes.

A fortunate genomic peculiarity of PCC 7942 helped us in the complex task of identifying xenologous genes. We used the scarcity of a highly iterated palindrome (5'-GCGATCGC-3') designated HIP1 [16] in combination with unusual codon usage, as a signal of recent *xenology*. HIP1 sequences are thought not to be mobile within the genome, but rather to form *in situ *through mutation [17]. HIP1 sequences are clearly overrepresented in the genome of PCC 7942 (7402 copies instead of 60 expected). The number of HIP1 sequences among ORFs in PCC 7942 ranges from 0 to 20 and their distribution is apparently random (Figure 2). As expected for a random process, the number of copies of HIP1 per gene is related to gene size (Figure 3), with some outstanding cases of ORFs having too few copies of HIP1 relative to their length.

It can be hypothesized that genes recently acquired by HGT (particularly from non-cyanobacterial species) would lack the HIP1 motif. By the same reasoning, genes residing for a long time in the genome would accumulate more HIP1 copies than recent xenologous genes. However, the lack of HIP1 (or the small number of copies relative to gene size) might be due to selection against HIP1 for a number of obvious reasons, as indicated by the lack of HIP1 copies in rDNA and most of the ribosomal protein genes (data not shown).

An initial set of ORFs with relative few copies of HIP1 motifs was identified as follows. We first formed groups of ORFs according to their number of HIP1 motifs (0, 1, ... *n*). Then, for all such groups with more than 50 ORFs, we identified those ORFs with a size larger than 1standard deviation (1SD) of the ORF size distribution (Figure 3). Hereafter all these ORFs will be referred as HIP1^less^. Then, to discriminate between the two possibilities (*xenology *or natural selection against HIP1 motif) among the HIP1^less ^set, we looked at codon usage. A bias in codon usage among genes in a genome is sometimes related to translation efficiency. This is especially true in species with large population sizes where natural selection can have a larger influence in fine tuning highly expressed genes to match tRNA abundance in the cell [18]. An algorithm to measure codon bias among different gene classes was introduced by [19]. For instance, in prokaryotic genomes, genes that deviate strongly in codon usage from the average gene but are sufficiently similar to codon usage from ribosomal protein genes (and to other experimentally determined highly expressed genes like transcription factors and chaperone degradation proteins) are predicted as highly expressed (PHX). On the other hand, genes that differ from standard classes of highly expressed genes, and to the codon usage of the average gene in the genome, are predicted as putative alien (PA). We used the algorithm developed by [19] as a criterion to discriminate between xenologs and highly expressed genes among those in the HIP1^less ^set. Accordingly, we measured the average codon usage for all genes in the genome (B(F|C)) as a reference class of an average gene, and the average codon usage of ribosomal proteins (B(F|RP)) as a standard class of highly expressed genes.

However, B(F|X) where (X denotes C or RP gene classes) is affected by ORF size (smaller ORFs tend to give values closer to 1, see Additional File 1) making it difficult to compare the same measure (i.e., B(F|C)) among genes with different sizes. To correct for bias due to differences in ORF sizes, we plotted B(F|X) against the Log(gene length), adjusted a *ln *equation to data, and then we measured the differences between B(F|X) values (*y*_B(F|X)_) to their respective predicted values (*ŷ*_B(F|X)_). The calculated differences between observed and predicted values (*y*_B(F|X) _- *ŷ*_B(F|X)_) were then used as a measure of deviation of codon usage when compared to the codon usage of an average gene in the genome (*y*_B(F|C) _- *ŷ*_B(F|C)_), or the codon usage of highly expressed genes (*y*_B(F|RP) _- *ŷ*_B(F|RP)_) (see Additional File 1).

Then, for all genes belonging to the HIP1^less ^set, we defined as PA genes, those having a positive difference larger than one standard deviation (1SD) in both measures. This indicates difference to both, highly expressed proteins on the one hand, and on the other hand, to the average gene in the genome. We defined as PHX those having a positive difference larger than 1SD in B(F|C), and a negative difference smaller than -1SD in B(F|RP), which indicates close similarity to codon usage of highly expressed ribosomal proteins. An extra category was introduced, namely (PX) for proteins having a pattern similar to highly expressed genes. In summary, the three categories can be defined by the following relationships (where ΔC = (y_B(F|C) _- ŷ_B(F|C)_) and ΔRP = (y_B(F|RP) _- ŷ_B(F|RP)_)):

(1) PA=[ΔC>1 SD C] AND [ΔRP>1 SD RP];

(2) $$\begin{array}{l} PX=[\Delta \text{C}>\bar{\text{X}}\text{ C}]\;AND\;[\Delta \text{RP}<-1\text{ SD RP}]\\ OR\\ {}[\Delta \text{C}>1\text{SD C}]\;AND\;[\Delta \text{RP}-\bar{\text{X}}\text{ RP}]; \end{array}$$

**Figure 4:**
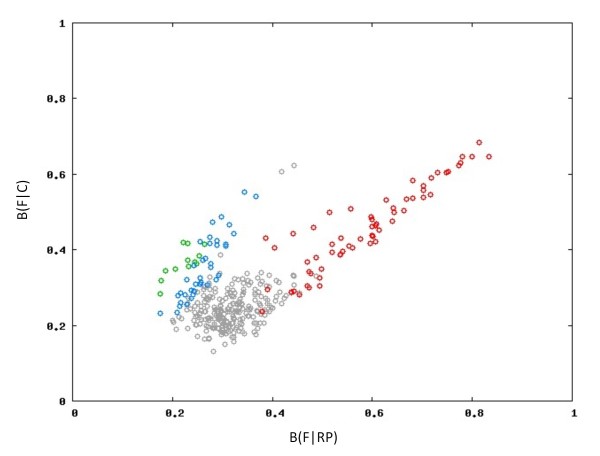
**Identification of putative alien (PA), highly expressed (PHX) and moderately highly expressed (PX) genes**. For each gene belonging to the HIP1^less ^set (having to few HIP1 motifs for its size) we calculated the codon usage bias against two set of genes: a) the mean codon usage of all genes in the genome B(F|C); and b) the codon usage of ribosomal proteins B(F|RP) which are expected to be highly expressed. Genes with a codon usage suggesting a high (PX) and very high (PHX) level of expression are shown in blue and green respectively. Genes with a codon usage suggesting a xenolog origin (PA) are shown in red. The rest of the genes belonging to the HIP1^less ^set that didn't meet the criteria to be included in PA, PX or PHX sets are shown in gray.

(3) PHX=[ΔC>1 SD C] AND [ΔRP<−1 SD RP];

By using these criteria we identified 60 genes as putative alien (PA), 38 showing a codon usage similar to genes with high levels of expression (PX), and 12 cases of putative highly expressed genes (PHX). A plot of B(F|RP) against B(F|C) illustrates the differences among these gene sets (Figure 4), where red, blue and green colors stand for PA, PX and PHX respectively (gray dots indicate genes from the HIP1^less ^set that didn't meet any of the three previous criteria). Clearly, those genes classified as highly expressed (PX and PHX) participate in important functions in the cell (Table 1).

**Table 1:** Protein products of genes classified as highly expressed (PHX) and moderately highly expressed (PX) according to codon usage.

Gen name*	length (bp)	Product	G+C desviation (SD)	N of HIP1 motifs
**Synpcc7942_0260**	824	ATPase	0	3
**Synpcc7942_0442**	497	ammonium transporter	0	1
**Synpcc7942_0656**	461	photosystem II 44 kDa subunit reaction center protein	0	0
**Synpcc7942_0697**	508	photosystem II core light harvesting protein	0	1
**Synpcc7942_0885**	694	elongation factor G	0	1
**Synpcc7942_0977**	333	Phosphoribulokinase	0	0
**Synpcc7942_1096**	456	thiamine biosynthesis protein ThiC	0	1
**Synpcc7942_1231**	324	apocytochrome f	0	0
**Synpcc7942_2048**	734	photosystem I P700 chlorophyll a apoprotein A2	0	2
**Synpcc7942_2049**	763	photosystem I P700 chlorophyll a apoprotein A1	0	1
**Synpcc7942_2463**	417	S-adenosylmethionine synthetase	0	0
**Synpcc7942_2468**	634	molecular chaperone DnaK	0	0
Synpcc7942_0143	326	pyruvate/2-oxoglutarate dehydrogenase (E1) component	0	0
Synpcc7942_0169	723	glutamate-ammonia ligase, glutamine synthetase type III	0	2
Synpcc7942_0282	427	serine hydroxymethyltransferase	0	1
Synpcc7942_0297	613	FtsH peptidase	0	2
Synpcc7942_0328	705	phycobilisome core-membrane linker polypeptide	0	1
Synpcc7942_0336	505	F0F1 ATP synthase subunit alpha	0	2
Synpcc7942_0385	457	geranylgeranyl reductase	0	2
Synpcc7942_0424	360	photosystem q(b) protein	0	0
Synpcc7942_0479	604	GTP-binding protein LepA	0	3
Synpcc7942_0538	668	Transketolase	0	3
Synpcc7942_0645	453	glutamate-1-semialdehyde aminotransferase	0	1
Synpcc7942_0685	555	chaperonin GroEL	0	2
Synpcc7942_0694	307	30S ribosomal protein S1	0	0
Synpcc7942_0734	624	hypothetical protein	0	2
Synpcc7942_0884	409	elongation factor Tu	0	1
Synpcc7942_0942	630	FtsH peptidase	0	1
Synpcc7942_0978	403	ferredoxin-NADP oxidoreductase	0	0
Synpcc7942_1426	472	ribulose bisophosphate carboxylase	0	2
Synpcc7942_1439	533	NAD(P)H-quinone oxidoreductase subunit 4	0	0
Synpcc7942_1443	357	fructose-1,6-bisphosphate aldolase	0	0
Synpcc7942_1463	543	Porin	0	1
Synpcc7942_1464	531	Porin	0	0
Synpcc7942_1522	1100	DNA-directed RNA polymerase subunit beta	0	1
Synpcc7942_1523	624	DNA-directed RNA polymerase subunit gamma	0	0
Synpcc7942_1524	1318	DNA-directed RNA polymerase subunit beta'	0	4
Synpcc7942_1525	597	GTP-binding protein TypA	0	1
Synpcc7942_1607	518	porin; major outer membrane protein	0	1
Synpcc7942_1719	475	isocitrate dehydrogenase	0	2
Synpcc7942_1782	430	threonine synthase	0	1
Synpcc7942_1907	358	magnesium-protoporphyrin IX monomethyl ester cyclase	0	0
Synpcc7942_2091	612	NAD(P)H-quinone oxidoreductase subunit F	0	5
Synpcc7942_2093	430	CO2 hydration protein	0	1
Synpcc7942_2156	473	L-glutamine synthetase	0	1
Synpcc7942_2160	384	alanine-glyoxylate aminotransferase	0	0
Synpcc7942_2313	544	chaperonin GroEL	0	1
Synpcc7942_2315	484	F0F1 ATP synthase subunit beta	0	1
Synpcc7942_2444	337	phosphate binding protein	0	0
Synpcc7942_2524	474	trigger factor	0	2

*PHX genes are shown in black, PX otherwise.

#### Unusual phylogenetic relationship

Another method to detect xenologous genes seeks for sequences having unexpected phylogenetic relationships. We carried out BLAST searches of all proteins coded in PCC 7942 against the Genbank non-redundant (nr) database. Then, we recorded whether or not the BLAST best-hit belonged to cyanobacteria. We also downloaded all best matches up to 10 sequences and, for all set of homologous proteins having at least four members, we reconstructed Neighbor-Joining trees with 100 bootstrap replicates. For all cases where it was possible to identify a unique nearest neighbor to the sequence from PCC 7942 (meaning that there is a partition that includes both sequences with the exclusion of any other OTU) with a bootstrap value over 50, we recorded whether it belonged to a cyanobacterial species.

Conflicting data from BLAST best-hit and Neighbor-Joining trees was treated as follows. As it is known that the BLAST best-hit is often not the nearest neighbor [20], we identified first all sequences having a nearest neighbor outside cyanobacteria according to Neighbor-Joining trees. Then, for those sequences for which it was not possible to use the phylogenetic criteria (because the immediate ancestral node of PCC 7942 protein did not share a unique co-descendent OTU; or the bootstrap value was lower than 50; or because there were not at least four homologs in the database), we used the BLAST best-hit criteria to decide whether the query sequence was more similar to proteins belonging to non cyanobacterial species.

BLAST comparisons against NCBI nr database allowed us also to identify those proteins in PCC 7942 without homologs, the so called ORFans. Although it is possible that a proportion of ORFans were false gene predictions, the possibility that many were real genes acquired by HGT from bacteriophages could not be ruled out [21]. By manually inspecting the list of ORFans, we removed eleven proteins that failed to hit homologs above specified threshold due to its small size. Among them, there were two very small proteins from the cytochrome complex (*petL *and *petM*) and one from the photosynthetic reaction center (*psaM*). We have also identified two genes coding for proteins from phages (Synpcc7942_0717 and Synpcc7942_2141).

**Figure 5:**
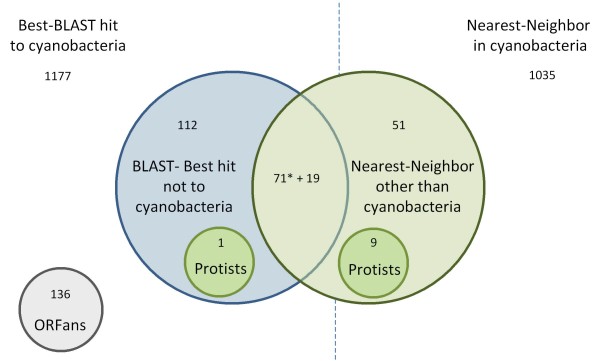
**Number of proteins in PCC 7942 with an unusual phylogenetic relationship**. For each protein coded in the genome of PCC 7942 we did blast searches against NCBI nr database to identify top scoring best hits. Then, for each protein having at least 4 hits we reconstructed a Neighbor-Joining tree (100 bootstrap replications). We then identified whether the nearest neighbor in the tree belonged to a cyanobacteria or not. When it was not possible to use the tree to identify the nearest neighbor (because the immediate ancestral node of PCC 7942 protein did not share a unique co-descendent OTU; or the bootstrap value was lower than 50; or because there were not at least four homologs in the database) we used the BLAST best hit criteria to decide whether or not the query protein has an unusual phylogenetic relationship. As shown, most proteins do not have its nearest homolog outside cyanobacteria (1177 + 1035 = 2212); however 253 proteins have an unusual phylogenetic relationship (10 of them to protists endowed with plastids). *Number of proteins in which the phylum identified was the same with BLAST and with Neighbor-Joining.

**Figure 6:**
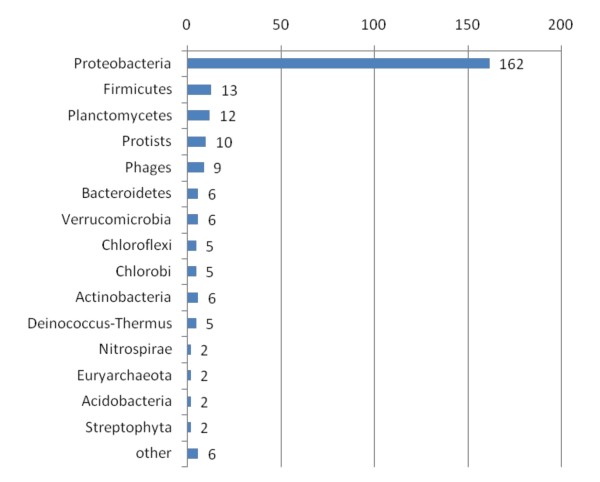
**Taxonomic classification of proteins having an unusual phylogenetic relationship**. Proteins having an unusual phylogenetic relationship are classified at phylum level. As shown, most of them are related to proteobacteria.

As expected, the vast majority of proteins in PCC 7942 were more similar to sequences from other cyanobacteria (2212 out of 2612 proteins). Nevertheless, we identified 253 proteins more similar to sequences from species other than cyanobacteria, plus 136 ORFans (Figure 5). Some of the hits to non-cyanobacterial species were to photosynthetic protists. Most of the similarities to non cyanobacterial species were to the phylum proteobacteria (Figure 6).

It should be kept in mind that having a nearest neighbor (or best BLAST hit) to a non-cyanobacterial species, does not necessarily mean that the gene in PCC 7942 was from HGT origin, because the opposite can also be true: that the gene in the non-cyanobacterial species originated by HGT from a cyanobacteria. Therefore, additional criteria should be taken into account to decide between the two possibilities.

#### G+C content

**Figure 7:**
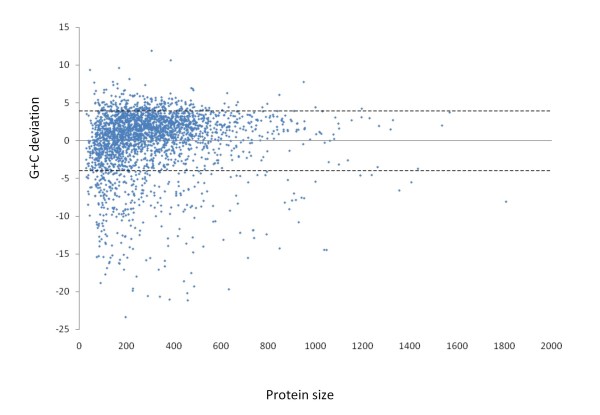
**Distribution of G+C content among ORFs in PCC 7942**. Each dot corresponds to an ORF in PCC 7942. The X axis indicated gene size translated into amino acids. Y axis indicated G+C content deviation from the mean. Mean and ± 1SD are shown by gray and doted lines respectively.

Although G+C deviation by itself is not a precise indicator of *xenology*, it can be useful in identifying recently acquired genes if it is used in combination with the other lines of evidence. In PCC 7942 there are 174 and 317 genes with a G+C content above, and below 1SD from the average, respectively (Figure 7).

#### Identification of xenologous genes by the combination of multiple approaches

**Figure 8:**
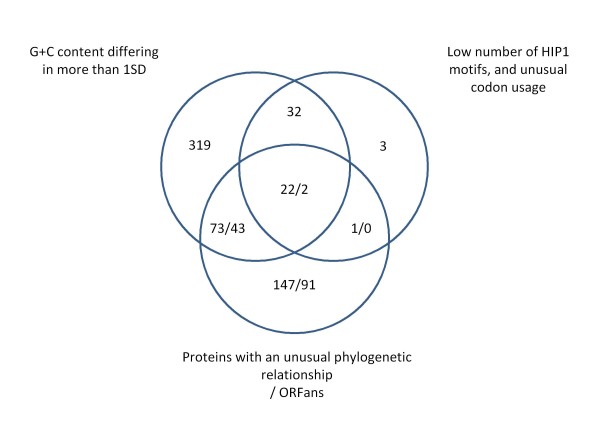
**Identification of xenologs in PCC 7942 by combining different sources of information**. Cases of horizontally transferred genes into the genome of PCC 7942 were identified by having at least two of the following properties: G+C content deviating more than 1 SD from the mean value of all genes; low number of HIP1 motifs relative to gene length well as an unusual codon usage (PA genes); and an unusual phylogenetic relationship. ORFan genes were also considered.

No single method is expected to identify all cases of HGT. For instance, composition based methods are biased towards the detection of relatively recent cases of HTG [22]. On the other hand, phylogenetic based methods detect ancient HGT events [23]. When looking for islands of variability in a genome where there are no genomes from different strains to compare, the best approach is to use a combination of methods, in order to avoid as much as possible the detection of false positives [24]. We compared the set of genes having an unusual G+C content, with those identified as PA (detected by atypical codon usage and low number of HIP1 motifs), and those having a nearest neighbor or best BLAST hit outside cyanobacteria. Those genes identified at least by two of the three methods were assumed to be xenologs. As shown in Figure 8, we detected 128 such cases. We have repeated the analysis with different SD cutoff values for the identification of PA, PX and PHX genes, and for the identification of genes deviating in G+C content. To evaluate the performance of these different cutoffs we used as a control set 323 genes that have not been subject to HGT among cyanobacteria [11]. The usage of 1SD for both cutoffs seems to be the best compromise between xenolog detection and avoidance of likely false positives (see Additional File 2).

#### Genomic island databases

We also surveyed information on GIs provided by IslandPath [25] and IslandViewer [26] databases. IslandPath provides information on *i*) G+C content; *ii*) regions of dinucleotide bias; *iii*) presence of known mobility genes like integrases and transposases; and *iv*) localization of tRNAs (known to be proximal to recombination places in the chromosome). By looking at this information, we identified the following eight new *xenologs*: *a*) a phage integrase (Synpcc7942_0136); *b*) a small genetic island consisting of a phage integrase-like (Synpcc7942_1205) followed by eight genes (three of them previously classified as xenologs by our method) and flanked by two tRNA-Cys; and *c*) a transposase (Synpcc7942_1842) not previously detected by our method.

IslandViewer database uses a combination of methods to detect GIs [26]. However, due to the lack of genetic diversity among *S. elongatus *sequenced genomes, the only useful method was SIGI-HMM [27]. This method uses a Hidden Markov Model (HMM) on measures of codon usage to identify possible GIs. Two islands, comprising 18 genes, were detected by this method, one located between nucleotides 48930 to 59068, and the other from 751277 to 758324. Of the 18 genes included on these islands, only 6 were previously undetected by our method, these are two genes coding for PilA-like proteins (Synpcc7942_0048, Synpcc7942_0049), two hypothetical proteins (Synpcc7942_0757, Synpcc7942_0758), and two XRE family transcriptional regulators (Synpcc7942_0763, Synpcc7942_0764).

#### Operon structure of xenologous genes

We looked at the predicted operon structure of those genes classified as xenologs to see if other genes within the same predicted operon had one of the characteristics previously used to identify cases of HGT (i.e., G+C bias, PA and/or nearest neighbor outside cyanobacteria). ORFans and genes from phages were also considered. If this was indeed the case, these genes were classified also as xenologs. By this method, we identified 25 predicted operons in which all the genes in the operon (besides the predicted xenologous gene), had at least one of the characteristics used to identify xenology (and thus adding 33 such genes to the list). The exceptions were seven predicted operons in which the accompanying genes did not show any of the characteristics mentioned above. These seven cases could be attributed to errors in the operon prediction algorithm, false positives in the method used here to detect xenologs, or to truly chimerical operons (in which only some of the genes comprising the operon arrived by horizontal gene transfer).

#### Comparison against metagenomic sequences from a fresh-water microbialite

Islands of variability in genomes are also detected by comparison to metagenomic sequences [28]. The rationale is simple; regions of variability in the genome of interest will be underrepresented, while core genome regions will be more represented in metagenomic sequences. The presence of cyanobacteria from the order of Chroococcales was reported in two metagenomes from fresh-water microbialites (Río Mesquitez and Pozas Azules) in the desert of Cuatro Cienegas, México [29]. In particular, sequences from Chroococcales in the metagenome of Río Mesquitez were the second most represented group with almost 20% of the classified sequences.

**Figure 9:**
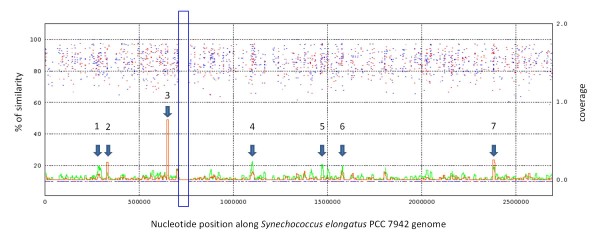
**Coverage of sequences from the metagenomes of two microbialites (Río Mesquitez and Pozas Azules) found in the region of Cuatro Cienegas along the genome of PCC 7942**. X axis indicates the nucleotide position along the PCC 7942 genome. Left axis indicates the percent of similarity of aligned metagenomic sequences to the genome of PCC 7942 (blue and red dots correspond to alignments to forward and reverse strands to *S. elongatus *DNA). The position of these alignments along the genome are also shown at the bottom of the figure to differentiate regions of coverage from non-coverage; right axis indicates a relative measure of coverage of sequences from the metagenomes on the genome of PCC 7942 (the green line at the bottom corresponds to the metagenome from Río Mesquites; while the red line to the metagenome from Pozas Azules). Coverage was calculated for windows of 10,000 nucleotides (sliding 500 nt.) as: (number of aligned nucleotides along the genome)/10,000. The blue rectangle indicates a large region of no coverage along the genome. Larger coverage peaks correspond mostly to genes classified as PX or PHX according to codon usage. The following genes contribute to the largest coverage peaks (1) FtsH peptidase (Synpcc7942_0297, PX); (2) F0F1 ATP synthase subunit alpha (Synpcc7942_0336, PX); (3) photosystem II 44 kDa subunit reaction center protein (Synpcc7942_0656, PHX); (4) ATPase, E1-E2 type (Synpcc7942_1082, PX), glycosyltransferase (Synpcc7942_1083), glycogen branching enzyme (Synpcc7942_1085, PX), ATPase (Synpcc7942_1089, PX); (5) protochlorophyllide reductase iron-sulfur ATP-binding protein (Synpcc7942_1419), light-independent protochlorophyllide reductase subunit N (Synpcc7942_1420, PX), putative carboxysome assembly protein (Synpcc7942_1421); (6) two component transcriptional regulator (Synpcc7942_1453); (7) F0F1 ATP synthase subunit beta (Synpcc7942_2315, PX).

Although the percentage of the genome of PCC 7942 that is covered by metagenomic sequences from Río Mesquitez and Pozas Azules was low (~1.5%), there was a large gap in coverage between approximately nucleotides 710,000 and 775,000 (Figure 9) suggesting the presence of an island of variability in this region. This GI coincides with one of the regions reported with SIGI-HMM algorithm (located between nucleotides 751277 to 758324), with the largest region of contiguous genes having a dinucleotide bias according to IslandPath database (comprising from gene Synpcc7946_0717 to Synpcc7942_0764), and with the largest contiguous region of likely xenologous genes detected here. Interestingly, high coverage regions corresponded to genes predicted to be highly expressed.

### Detection of conserved genes

Highly conserved genes comprise the other side of the coin when looking for deletable regions in a genome. Phylogenetic distribution of homologous sequences across genomes often follows a continuum, from genes present in just a single genome, to highly conserved genes present in a wide variety of lineages. This distribution has been described as consisting of three main clusters, namely, a small core of highly conserved genes, a larger shell of moderately conserved genes, and a much larger cloud of genes conserved among few lineages [30]. Highly conserved genes tend to be part of the so called core genome or *paleome *(the set of genes necessary for maintaining and replicating the cell), and genes with narrower phylogenetic distribution tend to be part of the *cenome *(genes necessary for living in a particular environment) [31].

To identify the paleome genes in PCC 7942 we considered the following lines of evidence, a) genes in PCC 7942 belonging to the cyanobacterial genome core CyOG database [32]; b) genes with homologs in at least 34 out of the 35 completely sequenced cyanobacterial genomes (where each homolog must have an alignment of at least 75% similarity to the query sequence); and c) genes with reciprocal best hits to the genome of the chromatophore of *Paulinella chromatophora *[33] and to the genome of *Prochlorococcus marinus *SS120 [34].

By using these four datasets we can classify genes from the core genome in different categories. For instance, genes belonging to CyOG database and genes conserved in at least 34 of the 35 genomes studied here, very likely are essential for any cyanobacterium. However, due to the evolutionary distance among sequenced cyanobacteria, the set of genes in CyOG and conserved among the genomes studied here, is a minimal and certainly not enough to maintain a cell by themselves. Complementary to this, *P. marinus *SS120 has been proposed as a model for a minimum free-living photoautotrophic cell because it has one of the smallest genomes among cyanobacteria [34]. The comparison against this genome is informative about how far away is *S. elongatus *from a real free-living photoautotrophic bacteria with a reduced genome. The chromatophore of *P. chromatophora *has a very small genome with only ~800 protein coding genes and is phylogenetically related to *P. marinus *SS120. Although not a free living entity, genes retained in the chromathophore are likely to be important for the photoautotrophic life style [33]. The comparison against the chromatophore is indicative of the genes necessary to maintain a real non-free living photosynthetic cell.

**Figure 10:**
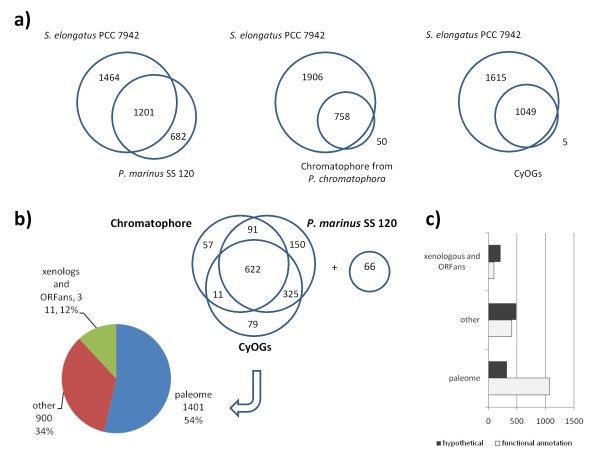
**Conserved genes in PCC 7942**. a) Number of best reciprocal BLAST hits between PCC 7942 and each of the following genomes: *P. marinus *SS 120, the chromatophore from *P. chromatophora *and the set of cyanobacterial conserved genes from CyOGs database; b) the set of conserved genes in PCC 7942 is defined as the union of the tree above datasets, plus those conserved in 34 out of 35 genomes (this last comparison added 66 genes to the dataset). Overall, the set of conserved genes comprises 54% of the genome of PCC 7942; c) the set of conserved genes (or paleome) is enriched in genes with functional annotation.

The number of genes in PCC 7942 belonging to the CyOG set, as well as those identified by best BLAST hits with the two models of minimal photoautotrophic genomes, are shown in Figure 10a. When looking at the intersection of these data, it turned out that most (622 genes) were shared between the three datasets (Figure 10b). In addition, there were 66 genes shared by at least 34 out of 35 genomes analyzed that were not present in any of the three previous comparisons. By pooling all four sets together, we defined the *paleome *as comprising 1,401 conserved genes. This correspond to ~54% of all protein coding genes in PCC 7942 (Figure 10b). It is interesting to note that the set of genes belonging to the *paleome *coincides with the number of genes of free-living prokaryotes with the smallest genomes (i.e., *Candidatus *Pelagibacter ubique with 1394 genes [35]; methylophilial bacteria HTCC2181 with 1377 genes [36]; *Ignicoccus hospitalis *KIN4/I with 1494 genes [37]). On the other hand, the set of genes likely to be xenologs correspond to ~12% of protein coding genes in the genome. As expected, xenologs and ORFans have the largest proportion of genes annotated as hypothetical proteins, while the *paleome *has the smallest one (Figure 10c).

### Signal transduction systems

**Figure 11:**
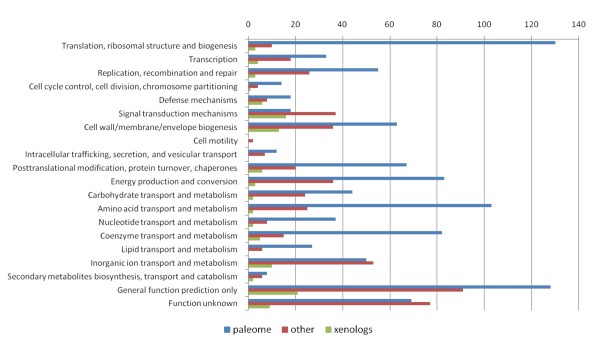
**Functional classification of xenologs and conserved genes in PCC 7942**. Following COG database 1,921 genes from PCC 7942 are classified into functional categories. Of them, 1,665 genes are classified in a single category. Here we show whether these genes are identified as core genes, xenologs or none of them. Xenologous genes are particularly abundant in the categories signal transduction mechanisms and cell wall/membrane/envelope biogenesis.

According to COG database, 1921 genes from PCC 7942 are classified in one or another functional category. Of them, 1665 are assigned to only one functional category. On Figure 11 we show the COG classification of these genes and whether they are likely xenologs, belong to the *paleome*, or neither of them. As shown, categories having more xenologs (besides 'General function prediction only') are 'Signal transduction mechanism' and 'Cell wall/membrane/envelope biogenesis'.

**Figure 12:**
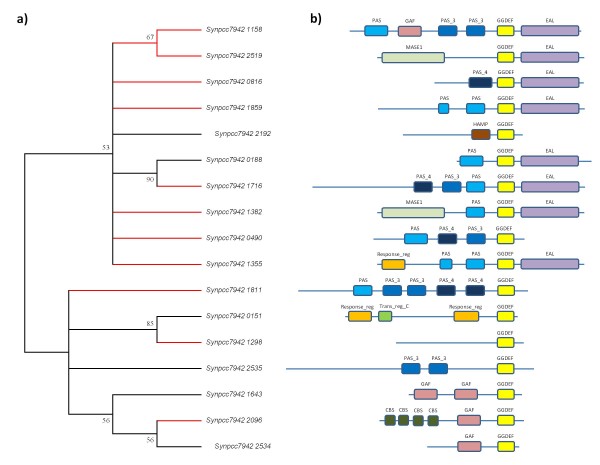
**The role of horizontal gene transfer in the evolution of sensory systems in PCC 7942**. Several proteins containing diguanylate cyclase and other domains involved in signal transduction mechanisms are predicted to be xenologs. Here we show a) a neighbor-joining tree reconstructed using the diguanylate-cyclase domain (86 sites, Poisson correction, 100 bootstrap replicates, branches with bootstraps below 50 are collapsed) from these proteins. Those proteins predicted to be xenologs are colored with red branches in the tree; b) domain structure of proteins following Pfam database [52].

Among genes belonging to 'Signal transductions mechanisms' category, it was particularly notable that, out of 17 genes having a diguanylate cyclase domain, 11 were predicted to be xenologs (Figure 12). As suggested by domains present in these proteins, they might participate in two component signal transduction systems. For instance GGDEF and EAL domains are involved in the turnover of cyclic-di-GMP in vivo [38]. On the other hand, PAS domains were found to act as sensors for light and oxygen in signal transduction systems [39]. Signal receiver domains [40] and GAF domains involved in regulation of cGMP [41] as well as the integral membrane sensory domain MASE1 [42] were also found. These data suggest that PCC 7942 could have acquired many environmental sensing capabilities through HGT.

### Horizontally transferred genes from central metabolism

We have found a series of horizontally transferred genes that participate in important metabolic roles within the cell. These include genes from oxidative phosphorylation, chlorophyll metabolism and starch and sucrose metabolism, among others (Table 2). Some of the transfers are clearly from non cyanobacterial species to *S. elongatus*, like in the case of perosamine synthetase. In other cases, the transfer seems to be among different species of cyanobacteria, like in the case of genes participating in oxidative phosphorylation where *S. elongatus *genes branches with genes from *Cyanothece *sp. (data not shown). In any case, it is noteworthy that genes that participate in central functions in *S. elongatus *arrived by horizontal gene transfer. Clearly, these genes are not candidates to be deleted from the genome.

**Table 2:** Horizontally transferred genes participating in metabolic roles in the cell.

Gene	Product	General function
Synpcc7942_0056	perosamine synthetase	Antibiotic biosynthesis
Synpcc7942_0184	putative phosphate permease	Transport
Synpcc7942_1007	fumarate hydratase	Citrate cycle
Synpcc7942_1398	cellulose synthase (UDP-forming)	Starch and sucrose metabolism
Synpcc7942_1468	putative monovalent cation/H+ antiporter subunit B	Oxidative phosphorylation
Synpcc7942_1473	putative monovalent cation/H+ antiporter subunit D	Oxidative phosphorylation
Synpcc7942_1474	putative monovalent cation/H+ antiporter subunit C	Oxidative phosphorylation
Synpcc7942_1851	ferredoxin--nitrite reductase	Nitrogen metabolism
Synpcc7942_1852	precorrin-8X methylmutase	Chlorophyll metabolism
Synpcc7942_1853	precorrin-2 C20-methyltransferase	Chlorophyll metabolism
Synpcc7942_1854	precorrin-3 methyltransferase	Chlorophyll metabolism
Synpcc7942_1855	cobyrinic acid a,c-diamide synthase	Chlorophyll metabolism
Synpcc7942_2151	cellulose synthase (UDP-forming)	Starch and sucrose metabolism
Synpcc7942_2238	glucose transport protein	Transport
Synpcc7942_2436	peptide methionine sulfoxide reductase	Repair Oxygen damage
Synpcc7942_2460	DNA-cytosine methyltransferase	DNA restriction-modification system

### False positives

18 genes were classified both as xenologs as well as belonging to the core set of genes, by the criteria used in this work. To decide whether these were true xenologs or false positives, we carried out a more thoughtful phylogenetic analysis. We reconstructed a phylogenetic tree using the first one hundred hits from BLAST against Genbank non redundant database, and we inspected the results visually. Xenology was still supported in ten cases, and seven of them seemed to be false positives (Table 3).

**Table 3:** Genes, detected both as xenologs and as core genes

Gene name	Protein product	Core genes*				Xenologs^¥^			Comment
		**1)**	**2)**	**3)**	**4)**	**A)**	**B)**	**C)**	

Synpcc7942_0056	perosamine synthetase			yes	29	-2		yes	Next to a genomic island, also detected by SIGI-HMM method, true prediction of xenology.
Synpcc7942_0587	valyl-tRNA synthetase	yes	yes	yes	35	-1		yes	No further phylogenetic evidence of xenology, false positive prediction of xenology.
Synpcc7942_1007	fumarate hydratase		yes	yes	35	-1	N-J	yes	The phylogenetic evidence suggests a true prediction of xenology.
Synpcc7942_1008	formyltetrahydrofolate deformylase			yes	33	-1			No phylogenetic evidence of xenology, false positive.
Synpcc7942_1064	type I restriction-modification		yes			1	Blast	yes	The phylogenetic evidence suggests a true prediction of xenology.
Synpcc7942_1080	K+ transporter Trk	yes	yes	yes	35	-2		yes	No further phylogenetic evidence of xenology, false positive prediction.
Synpcc7942_1081	hypothetical protein	yes	yes	yes	35	-1			No further phylogenetic evidence of xenology, false positive prediction.
Synpcc7942_1132	hypothetical protein			yes	4	-2		yes	No phylogenetic evidence of xenology, false positive prediction.
Synpcc7942_1660	potassium channel protein		yes		23	-1	Blast		No phylogenetic evidence of xenology, false positive prediction.
Synpcc7942_1851	ferredoxin--nitrite reductase				27	-2			Maby transferred among Cyanobacteria.
Synpcc7942_1852	precorrin-8X methylmutase		yes	yes	35	-1			Likely transferred among Cyanobacteria.
Synpcc7942_1853	cobalt-factor II C20-methyltransferase		yes	yes	35	-1			Likely related to alpha-proteobacteria.
Synpcc7942_1854	precorrin-3 methyltransferase		yes		26	-2	N-J		Closely related to alpha proteobacteria.
Synpcc7942_1855	cobyrinic acid a,c-diamide synthase		yes	yes	34	-2			Likely transferred among cyanobacteria.
Synpcc7942_1873	two component transcriptional regulator				35	-2			Likely transferred among cyanobacteria.
Synpcc7942_1887	mannosyltransferase		yes		8	-1	N-J		Likely transferred among cyanobacteria.
Synpcc7942_2188	isochorismate synthase	yes	yes	yes	33	1	N-J		Further phylogenetic analyses does not support evidence of xenology, false positive.
Synpcc7942_2436	peptide methionine sulfoxide reductase		yes		22	-1	N-J		Further phylogenetic evidence suggests a true prediction of xenology.

* Criteria to detect coregenes. For each gene, it is shown if it is conserved in 1) chromatophore of *P. chromathophora*; 2) *P. marinus *SS 120; 3) CyOGs database; and 4) number of cyanobacterial genomes with homologous genes. ^¥^Criteria to detect xenologs. A) G+C content in number of SD from the mean; B) unusual phylogenetic relationship by BLAST or Neighbor-joining; and C) Putative Alien (PA) genes.

## Conclusions

### Blueprint for a reduced photoautotrophic cell

**Figure 13:**
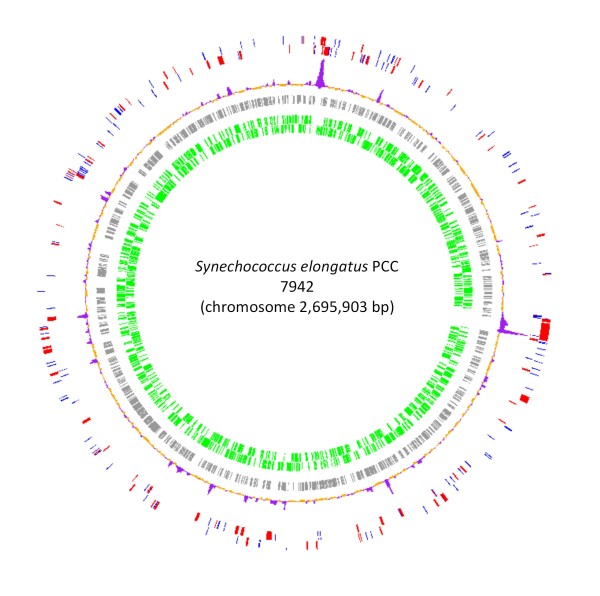
**Genomic islands and conserved genes in PCC 7942**. Starting from the outermost circle: First two circles: xenologs and ORFans are shown in red and blue respectively (both DNA strands are shown); third circle: regions of atypical three nucleotide composition (χ ^2 ^analyses, see methods) are shown as purple peaks; fourth circle: genes not classified as xenologs nor core genes are shown in gray (for simplicity both chains of DNA are shown as one); fifth circle, core genes in green (both DNA strands are shown). The genome was drawn using Genomeviz [53].

We present an analysis of conserved and variable regions in the genome of the cyanobacteria *Synechococcus elongatus*, strain PCC 7942. In Figure 13 we show the localization of these regions along the genome. The complete list of conserved and variable genes is shown in Additional File 3. Our results can be instrumental to test the essentiality/dispensability of genes in PCC 7942 by experimental approaches in the pursuit of a streamlined photoautotrophic platform.

Conserved genes were detected by combining the results of four analysis: (a) by looking at homologous in a dataset of 35 complete cyanobacterial genomes, and asking for at least 75% alignment coverage of the query sequence; (b) by looking for conserved proteins by reciprocal best BLAST hit with the reduced genomes of *P. marinus *SS120, and the chromatophore of *P. chromatophora*; and (c) by identification of proteins of PCC 7942 belonging to the set of conserved CyOGs [32]. As expected, a large fraction of genes (~45%) overlapped in the four datasets. These genes are likely to be essential under a broad spectrum of environmental conditions.

On the other hand, variable regions were detected by a combination of methods that in isolation might not offer strong evidence of xenology, but when combined, we believe them to have high predictive power to identify cases of HGT. Our analysis exemplifies how to detect GI in a phylogenetically isolated bacterial genome. In particular, the existence of HIP1 motifs facilitated the identification of recently acquired genes. Nevertheless, some bias (on the conservative side) in detection of xenologs was expected because the identification of genes with unusual phylogenetic relationships was designed to search for genes not belonging to cyanobacteria.

By eliminating dispensable genes, a simpler and smaller genome might be engineered that could be used as a chassis for diverse biotechnological applications. Although there is no guarantee that all genes in GI will be deletable without detrimental effects, by looking at their annotated functions it is possible to have a clue of their role in the cell. The elimination of genes that might destabilize the genome by introducing undesired mutations, like integrases and transposases, is one of the first obvious steps in this direction. Then, deletion experiments should be directed towards large GIs. If some of the proposed deletions have an effect on fitness, it would be necessary to allow for compensatory evolution to restore the system to a new equilibrium before experimentally attempting new deletions [43]. It also has to be noted that not all genes arrived by HGT are candidates to be deleted from the genome, as exemplified by most of the genes in Table 2. The approach presented here can be further complemented by mechanistic models of subsystems of the cell (like metabolic models) to indicate new avenues to engineer simpler biological systems.

## Methods

### Phylogenomic analysis

The complete genomes from 35 cyanobacteria plus 6 almost complete genomes from WGS projects (also from cyanobacteria) were downloaded from NCBI (http://www.ncbi.nlm.nih.gov). These were: *Synechococcus *sp. CC9605, *Synechococcus *sp. CC9902, *Synechococcus *sp. CC9311, *Synechococcus *sp. RCC307, *Synechococcus *sp. WH 8102, *Synechococcus *sp. WH 7803, *Prochlorococcus marinus *str. MIT 9303, *Prochlorococcus marinus *str. MIT 9313, *Prochlorococcus marinus *subsp. *marinus *str. CCMP1375, *Prochlorococcus marinus *str. AS9601, *Prochlorococcus marinus *str. MIT 9515, *Prochlorococcus marinus *str. NATL1A, *Prochlorococcus marinus *str. MIT 9301, *Prochlorococcus marinus *str. MIT 9215, *Prochlorococcus marinus *str. MIT 9312, *Prochlorococcus marinus *str. MIT 9211, *Prochlorococcus marinus *subsp. *pastoris *str. CCMP1986, *Prochlorococcus marinus *str. NATL2A, *Synechococcus elongatus *PCC 6301, *Cyanothece *sp. PCC 7425, *Thermosynechococcus elongatus *BP-1, *Acaryochloris marina *MBIC11017, *Anabaena variabilis *ATCC 29413, *Nostoc *sp. PCC 7120, *Nostoc punctiforme *PCC 73102, *Trichodesmium erythraeum *IMS101, *Synechococcus *sp. PCC 7002, *Synechocystis *sp. PCC 6803, *Cyanothece *sp. PCC 7424, *Microcystis aeruginosa *NIES-843, *Cyanothece *sp. PCC 8801, *Cyanothece *sp. ATCC 51142, *Synechococcus *sp. JA-3-3Ab, *Synechococcus *sp. JA-2-3B'a(2-13), *Gloeobacter violaceus *PCC 7421. Putative orthologs among these genomes were identified by BLAST best-reciprocal hits [44]. From the set of putative orthologs identified (473 protein families), we selected those belonging to the set identified by [11], as it was suggested that these proteins are less prone to HGT during the evolution of cyanobacteria. The selected 231 protein families were aligned with MUSCLE software [45] and poorly aligned regions were trimmed with Gblocks [46] using default parameters. Then, aligned protein families were concatenated in a single matrix of 63,065 amino acids, and a Minimum-Evolution (using Poisson distance correction and 100 bootstrap replicas) and a Parsimony trees were reconstructed using MEGA4.0 software [47]. We also reconstructed a Maximum-Likelihood tree on de same data using TREE-PUZZLE software v5.2[54].

### Analysis of HIP1 motif

The complete genome from *Synechococcus elongatus *PCC 7942 (NC_007604) was downloaded from NCBI database and a script written in Perl language (available upon request) was used to identify the position of HIP1 motifs along the sequence and the number of motifs per ORFs.

### Unusual codon usage

Genes having unusual codon usage were identified by the algorithm of [19]. The algorithm works as follow: let *G *be a group of genes with average codon frequencies *g*(*x, y, z*) for the codon triplet (*x, y, z*), normalized for each amino acid codon family such that Σ *g*(*x, y, z*) = 1, where the sum extends over all codons (*x, y, z*) translated to amino acid *a*. Let *f*(*x, y, z*) indicate the average codon frequencies for the gene group *F*, normalized to 1 in each amino acid codon family. The codon usage difference of the gene family *F *relative to the gene family *G *(codon bias relative to *G*) is calculated by the formula

$$B(F|G)=\sum_{a}Pa(F)[\sum_{\left(x,y,z=a\right)}\left|\Delta CU\right|]$$

AND

ΔCU=f(x,y,z)−g(x,y,z)

Where {*P_a_*(*F*)} are the average amino acid frequencies of the genes of *F*. Then, *B*(*F*|*G*) refers as the codon bias of *F *with respect to *G*. We measured the codon bias of all protein coding genes in PCC 7942 with respect to the average codon frequencies of ribosomal proteins *B*(*F*|*RP*) and with respect to the mean codon usage of all ORFs in the genome *B*(*F*|*C*). All measurements were done using a script in Perl available upon request.

### Unusual phylogenetic relationship

BLAST searches of all amino acid sequences encoded in PCC 7942 were performed against Genbank non-redundant (nr) database and it was recorded whether the best hit corresponded to a cyanobacteria or not. We also downloaded the first 10 best hits and performed a Neighbor-Joining three on them using PHYLIP package [48]. We recorded whether the nearest neighbor OTU to the query sequence belonged to cyanobacteria or not. For the similarity to be significant, the bootstrap value of the node connecting both OTUs should be larger than 50. The analysis was carried out using Bioperl scripts [49] available upon request.

### Operon structure of xenologous genes

First we identified the operon structure of detected xenologous genes relying on the operon predictor algorithm provided by BioCyc database [50]. Then, we looked if other genes in the same operon as the detected xenologous ORFs showed at least one of the features used to detect xenologs previously (namely, biased G+C content; low number of HIP1 motifs and unusual codon usage; and/or best-hit or nearest neighbor to an organism other than cyanobacteria). If this was indeed the case, we classified the accompanying gene(s) also as xenologs.

### Comparison against metagenomic sequences

We compared the genome sequence of PCC 7942 against two metagenomes from fresh-water microbialites [29] using MUMmer 3.0 software [51].

### Core genome

To identify highly conserved sequences belonging to the core genome, several lines of evidence were followed. On the first place, best reciprocal BLAST hits were identified (independently) between: PCC 7942 and the chromatophore of *Paulinella chromatophora *[33] and between PCC 7942 and the genome of *Prochlorococcus marinus *SS120 [34]. Also, genes from PCC 7942 with homologs in at least 34 out of 35 complete genomes from cyanobacteria (having a coverage alignment of at least 75%) were also identified as belonging to the core. Finally, those sequences from PCC 7942 belonging to the set of CyOGs [32] were also classified as core genes.

### χ^2 ^*Analysis*

Regions of atypical nucleotide composition were identified by χ^2 ^analysis; the distribution of all 64 tri-nucleotides (3mers) was computed for the complete genome in all six reading frames, followed by the 3mer distribution in 5,000-bp windows. Windows overlapped by 500 bp. For each window, the χ^2 ^statistic on the difference between its 3mer content and that of the whole genome was computed. Peaks in Figure 12 indicate regions of atypical tri-nucleotide composition.

## List of abbreviations

GI: Genomic Island; HGT: Horizontal Gene Transfer; PA: Putative Alien; PHX: Putative Highly Expressed; PX: Similar to Highly Expressed; SD: Standard Deviation.

## Authors' contributions

LD carried comparative genome analysis and drafted the manuscript. CMGD carried phylogenetic analyses and help in drafting the manuscript. MPGB identified relevant molecules in PCC 7942 and helped in drafting the manuscript. JP made important conceptual contributions to the study. FC conceived the study made important contributions to draft the manuscript. AM conceived the study and made important contributions to draft the manuscript. All authors read an approved the final manuscript.

## Authors' information

LD: postoctoral specialist in bioinformatics; CMGD: posdoctoral specialist in bioinformatics; MPGB: posdoctoral specialist in molecular biology; JP: Associate Professor of Biochemistry and Molecular Biology; FC: Full Professor of Genetics; AM: Full Professor of Genetics.

## Supplementary Material

Additional file 1 **Differences of B(F|C) and B(F|RP) values (*y*_B(F|X)_), to their respective predicted values (*ŷ*_B(F|X)_) calculated by adjusting a *ln *equation to a chart of Log(gene length) versus B(F|C) and B(F|RP)**. Click here for file

Additional file 2 **Use of different SD values to identify xenologs**. Click here for file

Additional file 3 **List of all genes in PCC 7942 indicating whether they are xenologs or core genes**. Click here for file
