# Supplementary figures and images for: Blueprint for a minimal photoautotrophic cell: conserved and variable genes in Synechococcus elongatus PCC 7942
Source: BMC Genomics. 2011 Jan 12;12:25. doi: 10.1186/1471-2164-12-25 (PMC3025956; doi:10.1186/1471-2164-12-25)

## Slide 1
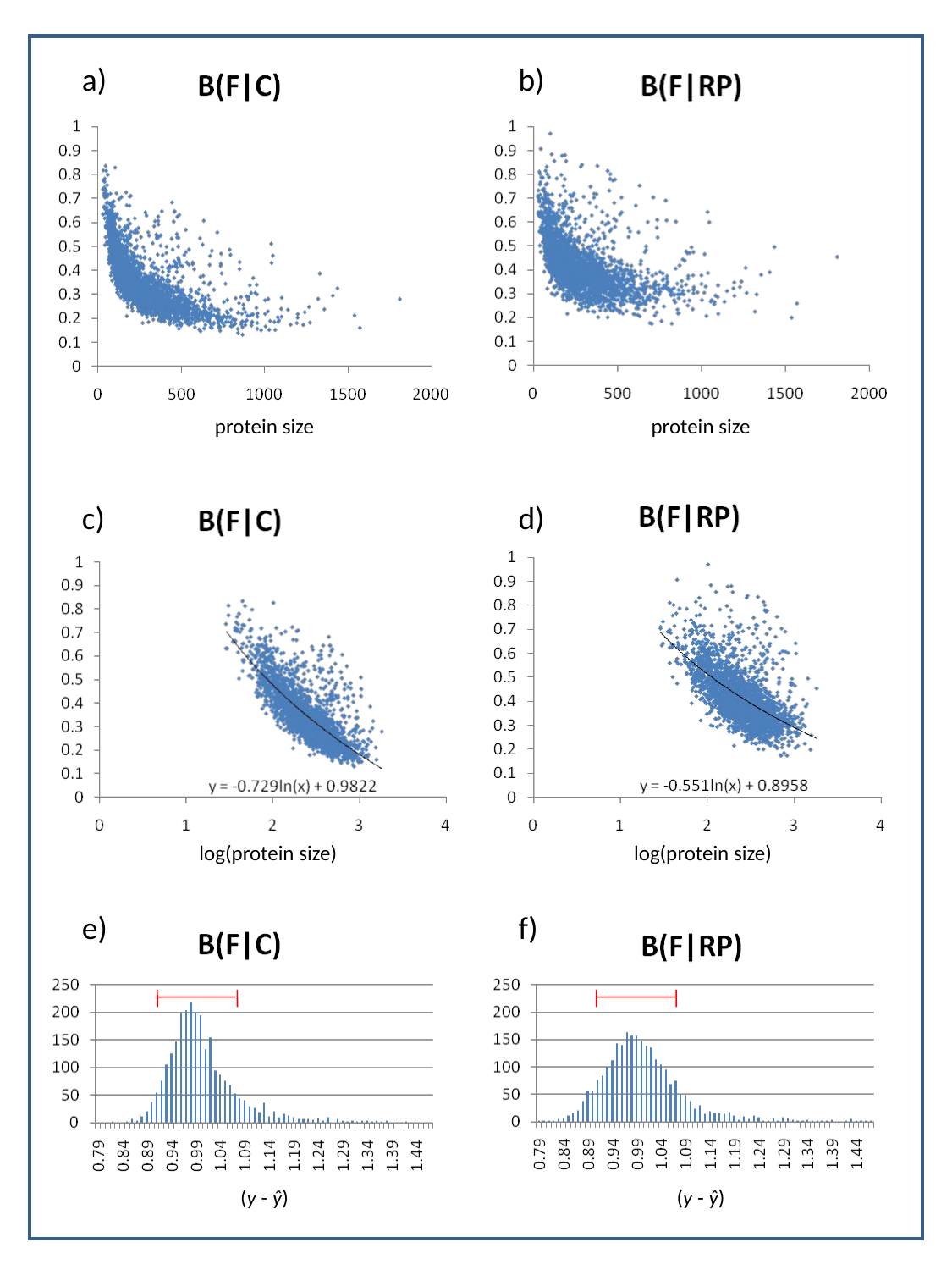

a)
b)
protein size
protein size
c)
d)
log(protein size)
log(protein size)
e)
f)
(y - ŷ)
(y - ŷ)

Supplement: Additional file 1 — Differences of B(F|C) and B(F|RP) values (yB(F|X)), to their respective predicted values (ŷB(F|X)) calculated by adjusting a ln equation to a chart of Log(gene length) versus B(F|C) and B(F|RP). [file 1471-2164-12-25-S1.PPT]
