# Supplementary material for: Blueprint for a minimal photoautotrophic cell: conserved and variable genes in Synechococcus elongatus PCC 7942
Source: BMC Genomics. 2011 Jan 12;12:25. doi: 10.1186/1471-2164-12-25 (PMC3025956; doi:10.1186/1471-2164-12-25)
Supplement: Additional file 2 — Use of different SD values to identify xenologs. [file 1471-2164-12-25-S2.DOC]

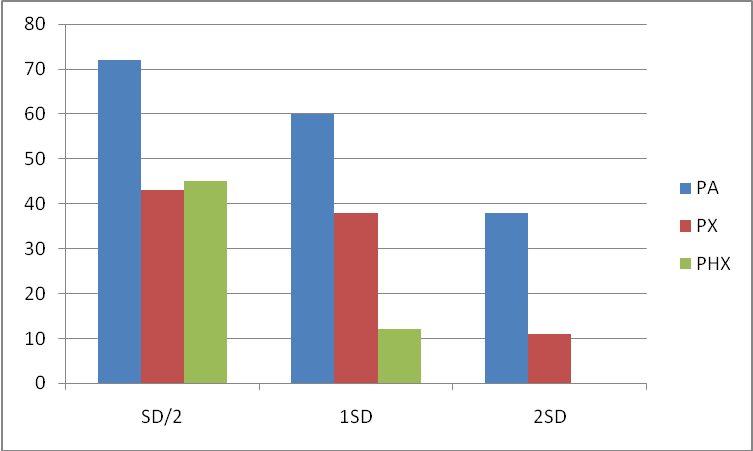


Figure S1. Number of detected PA, PX and PHX genes under different SD values (SD/2, 1SD and 2SD) in formula 1, 2 and 3 (see text).


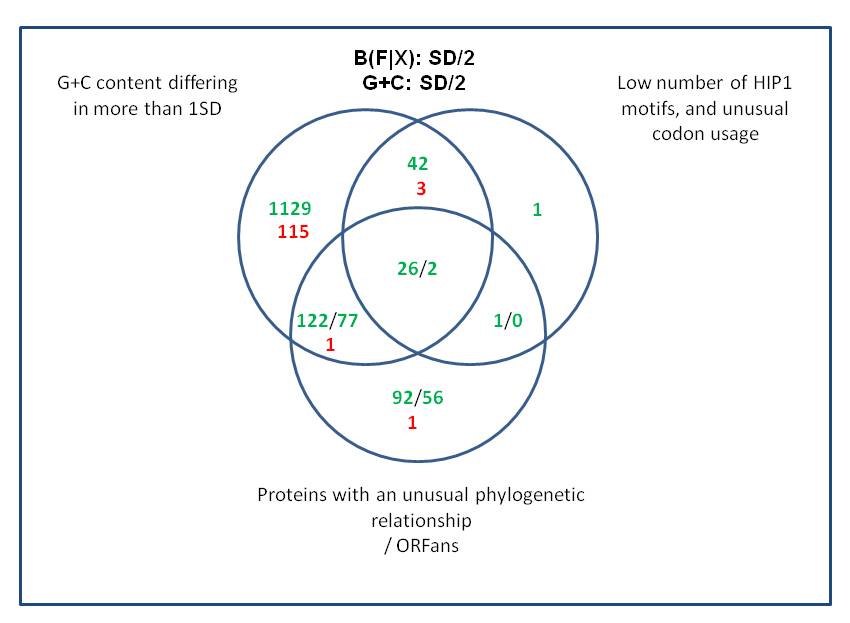


Figure S2. Number of xenolog ousgenes detected when using a cutoff value of SD/2 for formula 1, 2 and 3 and for G+C content. In red is the number of likely false positives (see text) among those detected in green.


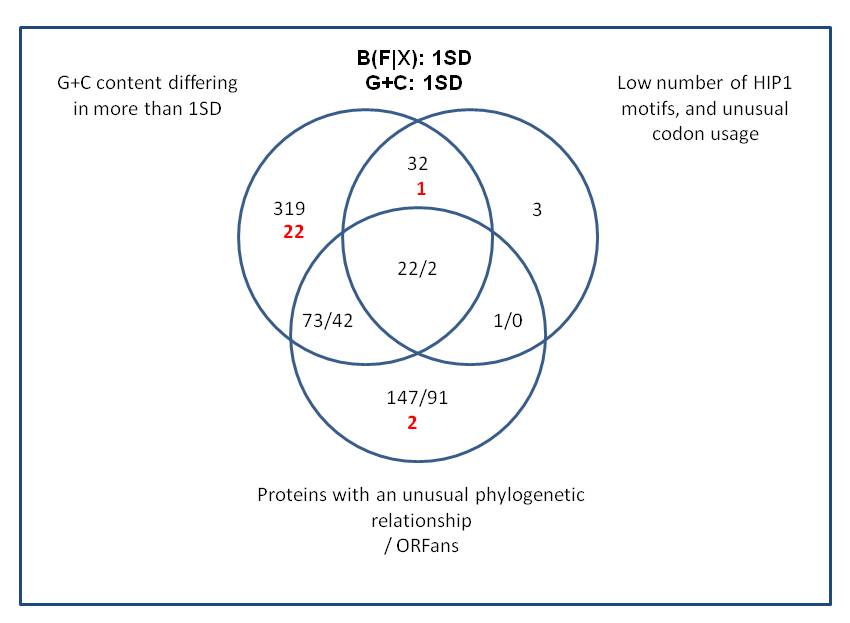


Figure S3. Number of xenologous genes detected when using a cutoff value of SD for formula 1, 2 and 3 and for G+C content. In red is the number of likely false positives (see text) among those detected in green.


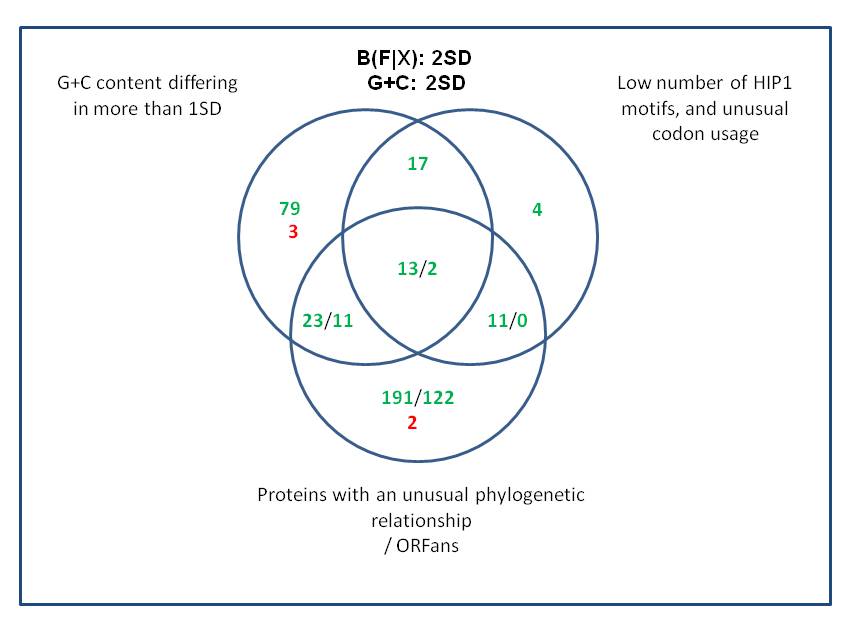


Figure S4. Number of xenologous genes detected when using a cutoff value of 2SD for formula 1, 2 and 3 and for G+C content. In red is the number of likely false positives (see text) among those detected in green.


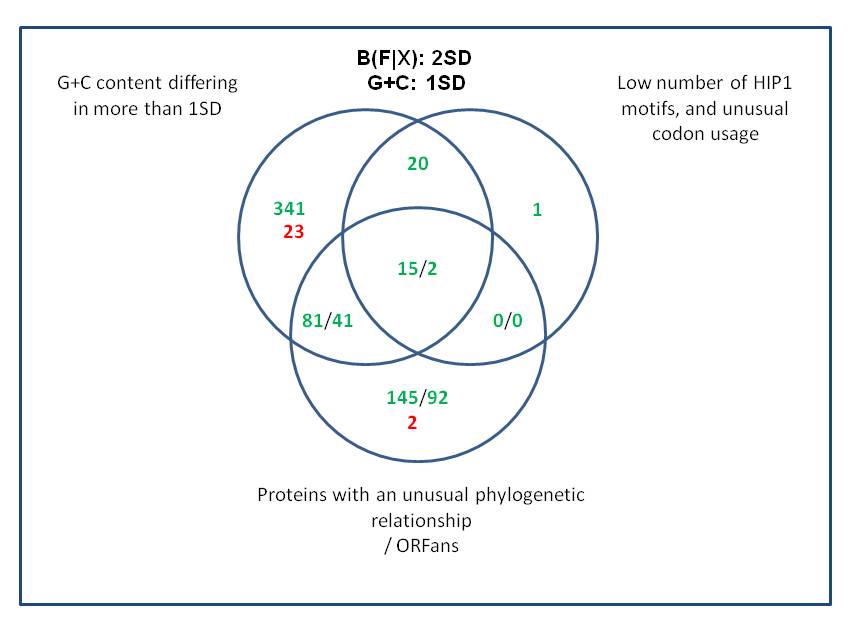


Figure S5. Number of xenologous genes detected when using a cutoff value of 2SD for formula 1, 2 and 3 and 1SD for G+C content. In red is the number of likely false positives (see text) among those detected in green.

|  | A  B(F|X): SD/2, G+C: SD/2 | B  B(F|X): 1SD, G+C: 1SD | C  B(F|X): 2SD, G+C: 2SD | D  B(F|X): 2SD, G+C: 1SD |
| --- | --- | --- | --- | --- |
| Xenologs | 270 | 170 | 77 | 159 |
| Set of 323 genes not suffering from HGT (false positives) | 5 | 1 | 0 | 0 |

Table 1S. Summary of the number of xenologs and likely false positives for four different combination of cutoff SD values for formula 1, 2, and 3 and for deviation in G+C content. Te cutoff value used in the analysis is shown in gray.
